# Oviductal Oxygen Homeostasis in Patients with Uterine Myoma: Correlation between Hypoxia and Telocytes

**DOI:** 10.3390/ijms23116155

**Published:** 2022-05-31

**Authors:** Anna Wrona, Veronika Aleksandrovych, Tomasz Bereza, Paweł Basta, Anna Gil, Magdalena Ulatowska-Białas, Małgorzata Mazur-Laskowska, Kazimierz Pityński, Krzysztof Gil

**Affiliations:** 1 Gynecology and Obstetrics Ward with Gynecologic Oncology Subdivision, J. Śniadecki’s Specialistic Hospital, 33-300 Nowy Sącz, Poland; annawrona788@gmail.com; 2 Department of Pathophysiology, Jagiellonian University Medical College, 31-121 Krakow, Poland; v.aleksandrovych@uj.edu.pl; 3 Department of Anatomy, Jagiellonian University Medical College, 31-034 Krakow, Poland; tomasz.1.bereza@uj.edu.pl (T.B.); anna.m.gil@doctoral.uj.edu.pl (A.G.); 4 Department of Gynecology and Oncology, Jagiellonian University Medical College, 30-688 Krakow, Poland; pawel.basta@uj.edu.pl (P.B.); kazimierz.pitynski@uj.edu.pl (K.P.); 5 Department of Pathomorphology, Jagiellonian University Medical College, 31-531 Krakow, Poland; magdalena.bialas@uj.edu.pl; 6 Diagnostic Department of University Hospital, Jakubowskiego 2 St., 30-688 Kraków, Poland; mmazur@su.krakow.pl

**Keywords:** hypoxia, angiogenesis, uterine myoma, oviduct, telocytes, VEGF

## Abstract

Oxygen balance is crucial for angiogenesis, immunity, and tissue repair. The human oviduct is essential for reproductive function, and any imbalance in homeostasis leads to fertility disturbances and might be a reason for ectopic pregnancy development. Uterine myoma is a widespread benign tumour, which is often accompanied by infertility. Telocytes have been discussed in the contexts of motility, fibrosis development, and angiogenesis. We observed the oviducts from patients with and without uterine myoma, comparing the expression of HIF-1, HO, VEGF and its receptor, NOS, oestrogen, and progesterone receptors by immunolabeling. The myometrial and oviductal telocytes were also compared in both groups. Biochemical analyses were conducted for FSH, LH, AMH, sFlt, oestrogen, and progesterone in blood samples. Patients with uterine myoma have different expressions of sex steroid receptors and an increased number of telocytes. The decreasing VEFG expression was compensated by the rise in the HIF-1 and NOS expression. Blood biochemical analyses revealed a higher progesterone level and lower AMH in patients with uterine myoma. No differences in sFlt, FSH, and LF were observed. Uterine myoma impacts oviduct oxygen homeostasis and might cause fertility disturbances (uterine and oviductal infertility factors).

## 1. Introduction

**Figure 1:**
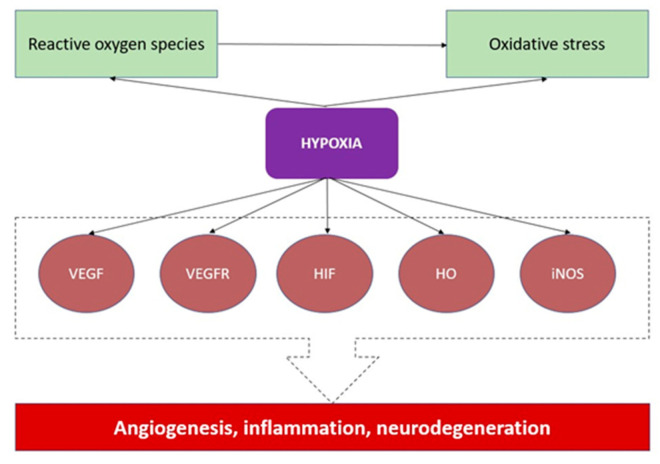
The influence of hypoxia in the contexts of angiogenic markers of homeostasis. Abbreviations: VEGF, vascular endothelial growth factor; VEGFR, vascular endothelial growth factor receptor; HIF, hypoxia-inducible factor; HO, heme oxygenase; iNOS, inducible nitric oxide synthase.

Oxygen homeostasis is essential for metabolism, tissue repair, regeneration, ageing, and normal physiology. Hypoxia is the presence of a lower-than-normal oxygen content and pressure in the cell [1]. A hypoxic environment can occur via the up- and downregulation of the gene expression and the secretion of chemokines and other biologically active substances. Hypoxia could switch on adaptative pathways, which might also be considered the required compounds of “oxidative stress” or “mitochondrial function”. They all form the background of disease pathogenesis in human and animal bodies (Figure 1) [2,3]. For instance, hypoxia upregulates mRNA in genes, including ALDOA (aldolase, fructose-bisphosphate A), ENO1 (alpha-enolase), LDHA (lactate dehydrogenase A), VEGF-A (vascular endothelial growth factor-A, also VEGF), PFKFB3 (6-Phosphofructo-2-Kinase/Fructose-2,6-Biphosphatase 3), and SLC2A1 (Solute Carrier Family 2 Member 1) [4]. In addition, hypoxia is often discussed in the context of the tumour microenvironment and inflammation [1,2] because the hypoxic local microenvironment stimulates the secretion of chemokines that impact nearby tissue. This phenomenon has already been described in oncological diseases [2,3,5]. Chronic hypoxia promotes tissue repair via fibrosis development [5], while “inflammatory hypoxia” stimulates the proliferation of neutrophils and monocytes and the activation of oxygen-consuming enzymes [6]. The oxygen gradient is essential for physiological immunological niches (placenta, bone marrow, lymph nodes, gastrointestinal tracts) and is crucial for hypoxia-inflammation signalling crosstalk [7,8,9]. Chronic inflammation as chronic hypoxia is a trigger for carcinogenesis [8,9,10]. The production of hypoxia-inducible factor (HIF)-1 has been induced by hypoxia, which also stimulates inducible nitric-oxide-synthase (iNOS) production. Intracellular nitrogen species (RNS) and reactive oxygen species (ROS) rise as a consequence, which results in DNA damage [10,11]. Kawanishi et al. stress that tumour-producing inflammation could induce nuclear factor-κB, resulting in iNOS-dependent DNA damage [10]. Meanwhile, immunity also indirectly correlates with the hypoxia level—HIF plays a role in regulating innate and adaptive immunity [7]. In macrophages, the expression of HIF-1 correlates with glycolysis, ATP generation, cytokine production, bacterial killing, and migration/invasion. In neutrophils, HIF-1 mostly correlates with killing bacteria [12]. Wobben et al. show that the HIF-1α protein is required for the adequate production of interferon-α and -β [13]. The differentiation of T-lymphocytes occurs upon HIF production [14], whereas, in B-lymphocytes, HIF also impacts survival, development, and antibody processing [7,15]. Moreover, Taylor et al. describe six anatomic-histological units with “physiological hypoxia”, which is characterised by the naturally low partial pressure of oxygen: the intestinal mucosa, the renal medulla, the bone marrow, the placenta, the retina, and the light zone of the germinal centres of lymph nodes [7]. Alpha-enolase, which is upregulated in hypoxia, is essential for cancer invasion and impacts the humoral and cellular immune response [16].

A prominent role of hypoxia is the regulation of angiogenesis, with or without interaction with other proangiogenic factors (VEGF, placental growth factor, fibroblast growth factor-2, platelet-derived growth factor) [17]. The vascular endothelial growth factor family and its receptors can be discussed in the context of female tubal infertility. Decreasing its expression might lead to embryo implantation in the oviduct, resulting in ectopic pregnancy development [18]. The expression of VEGF and its receptors is not equal in all parts of the oviduct. For instance, in the porcine oviduct, the expression of VEGFRA mRNA is higher in the ampulla, while fms-like tyrosine kinase-1 (Flt-1) mRNA, a receptor of VEGF, is higher in the isthmus. VEGFRA protein has the same tendency, but no difference in the expression of the receptor to this protein between the ampulla and isthmus has been reported. It is important to note that the expression of proteins and their receptors changes during the cycle, which stresses their role in oviductal physiology and fertility. López Albors et al. suggest that VEGR regulates the dynamics of oviductal fluid secretion and oviduct motility [19].

Telocytes (TCs) are interstitial cells with extremely high potency in forming homo- and heterocellular contacts with different types of cells. Previously, it was reported that they are more sensitive to local ischemia than other stromal cell types, such as fibroblasts, myofibroblasts, or mast cells [20]. Their role in the tumour microenvironment has been recently discussed [21]. Furthermore, oviductal telocytes might play a role in oviduct motility and contractility. They have been observed in patients with endometriosis [22], uterine myoma [23], and inflammatory gynaecologic disease [24]. Observing how uterine pathology correlates with oviductal and oxygen homeostasis is essential. Uterine myoma is accompanied by fibrosis development and the formation of a vascular capsule [4,25]. Avascular areas in the myometrium, affected by myoma, lead to hypoxia development, which, in turn, stimulates the production of angiogenic factors. Ishikawa et al. suggest this HIF-1α-mediated hypoxic response in uterine leiomyoma [4]. Zheng et al. hypothesised that TCs might suppress oxidative stress. These cells might produce VEGF, whose receptors’ activation (upon the binding of VEGF) stimulates angiogenesis via the induction of tube formation in vascular endothelial cells [20,26]. Meanwhile, TCs in the uterus and heart may release signalling molecules (interleukin −2, −6, −10, and −13, VEGF, epidermal growth factor (EGF), macrophage inflammatory protein 1α, macrophage inflammatory protein-2, monocyte chemoattractant protein 1, and growth-related oncogene/keratinocyte-derived chemokines) [21,26,27,28,29].

Our study aims to compare the expression of angiogenic factors in the oviducts and myometrium of patients affected and unaffected by uterine myoma. We also intend to check the correlation between telocytes and angiogenic markers to reveal their possible involvement in local homeostasis. The level of sex steroid hormones will be checked in blood samples and will also be compared, using results from our former studies [23,24].

## 2. Results

### 2.1. General Structure of the Human Oviduct and Myometrial Tissue

No difference in the histological structure of the human oviduct in patients with and without uterine myoma was revealed. The haematoxylin-and-eosin staining of the oviductal tissue showed all typical layers: the serosa (adventitia layer), the muscle layer (muscularis mucosa, consisting of an outer longitudinal layer and inner circular layer), and the mucosa. The focuses of myoma were characterised by the prevalence of collagen deposits and chaotic organisations (directions and crossing of fibres).

### 2.2. Expression of Proangiogenic Markers

The VEGF expression was higher in the myometrium from the control group than in the myoma’s foci. There was minimal VEGF expression in gross myomas, while it was slightly higher in small foci. However, in some areas in the myoma, close to the capsule, we observed islands of tissue with an increased expression of VEGF, compared to the central myoma. The highest growth factor expression was observed in areas of adjacent myometrium surrounding the myoma. Oviducts from patients with uterine myoma had less VEGF expression than the control group. We noted that VEGF was generally high in myometrium, compared to the oviducts in patients without myoma (Table 1).

The VEGF receptor expression in the oviduct tissue was high in both groups, with a slight predominance in patients without uterine myoma. We found lower VEGF receptor expression in healthy myometrium than in the myoma foci; in the adjacent myometrium, it was raised. In comparing the staining, we noted that it was lower in the myometrium surrounding myomatous nodes than in the uterus unaffected by myoma. In oviducts, the receptor expression was higher in the control, but only slightly declined in the study group.

**Figure 2:**
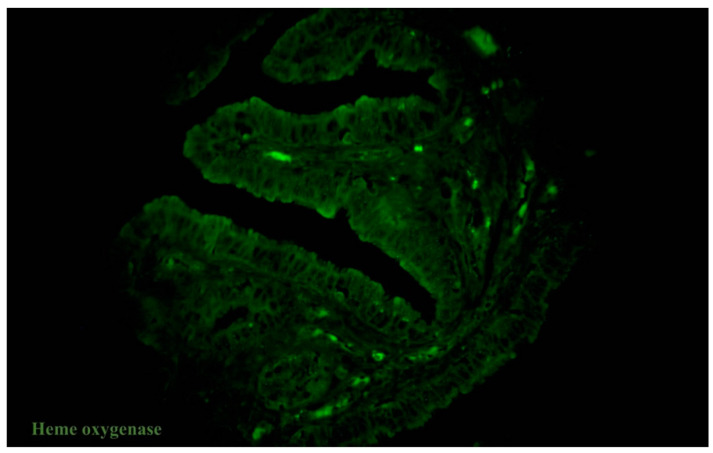
Tissue sample of human oviduct stained for HO-1 (green, Alexa Fluor 488) in the patient with uterine myoma. Total magnification: ×400.

We observed the changing expression of haem oxygenase-1 in oviducts, depending on the presence/absence of uterine myoma. The gross pattern of oviduct tissue from both groups showed that the HO-1 expression was higher in the control group. In this group, the HO-1 expression was higher in the tunica mucosa and submucosa compared to the muscular layer. However, the study group was characterised by the increased expression of HO-1 in the submucosa and low expression between the muscular fibres (Figure 2). The HO-1 expression was the highest in the healthy myometrium and declined in the myoma. We observed increased expression on the borders of myomatous nodes and in the areas of the surrounding myometrial tissue (but it was lower than in healthy myometrium). The pattern of expression also changed. There were lines accompanying muscle fibres in the control group, while, in the myomatous uterus, there were islands, and sometimes near the blood vessels.

In the tissue samples from the uterine myoma, the expression of HIF was very low, and, in some areas, it was almost absent. The myometrium from the control group had a medium level of HIF expression. The adjacent myometrium was characterised by increased expression: generally, it was similar to the healthy myometrium, but, in some areas, it was higher than in the unchanged myometrium. The HIF in the human oviducts from both groups was expressed less than in the myometrium. The oviductal tissue from the study group had a slightly higher expression of HIF than the control group.

### 2.3. Expression of NOS in the Human Oviduct

**Figure 3:**
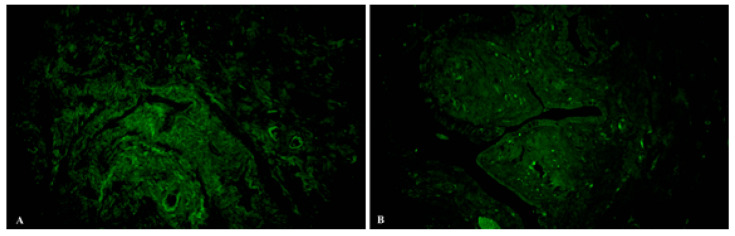
Samples of human oviduct stained for NOS (green, Alexa Fluor 488) in the control (**A**) and study (**B**) groups. Total magnification: ×200.

The expression of NOS was elevated in the control group, with domination in the muscular layer. In patients with uterine myoma, it declined, and mainly in the submucosal layer. The expression of NOS-positive cells and fibres between the muscles in the oviducts from the study group was lower than the control group (Figure 3).

### 2.4. Expression of Tubal and Uterine Telocytes

**Figure 4:**
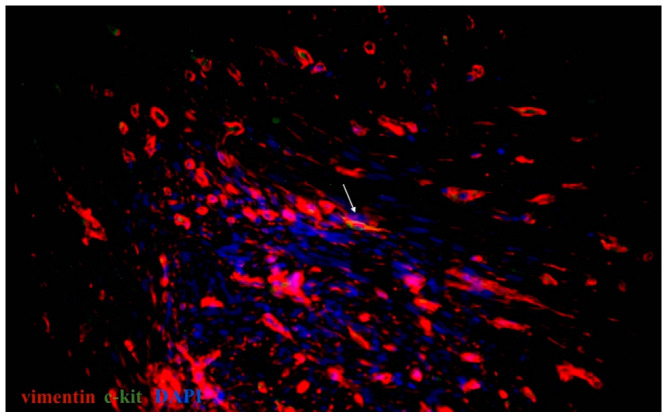
Double immunolabelling of tissue sample from uterine myoma stained for vimentin (red, Alexa Fluor 594) and c-kit (green, Alexa Fluor 488). Nuclei stained by DAPI. Doubly immunopositive cell with oval-shaped body and cellular prolongation identified as a telocyte (marked by an arrow). Total magnification: ×400.

**Figure 5:**
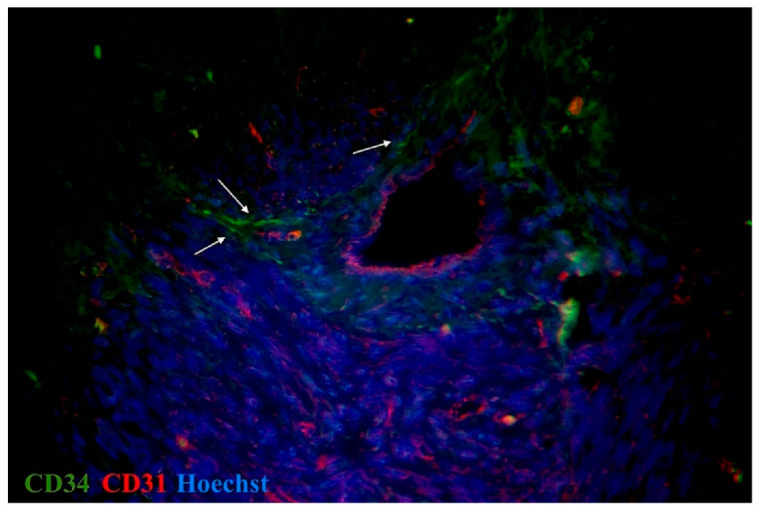
Double immunolabelling of human myometrium stained for CD31 (red, Alexa Fluor 594) and CD34 (green, Alexa Fluor 488) from control. Nuclei stained by Hoechst 33342. Doubly immunopositive cell with long cellular prolongation identified as telocytes (marked by arrows). Total magnification: ×400.

**Figure 6:**
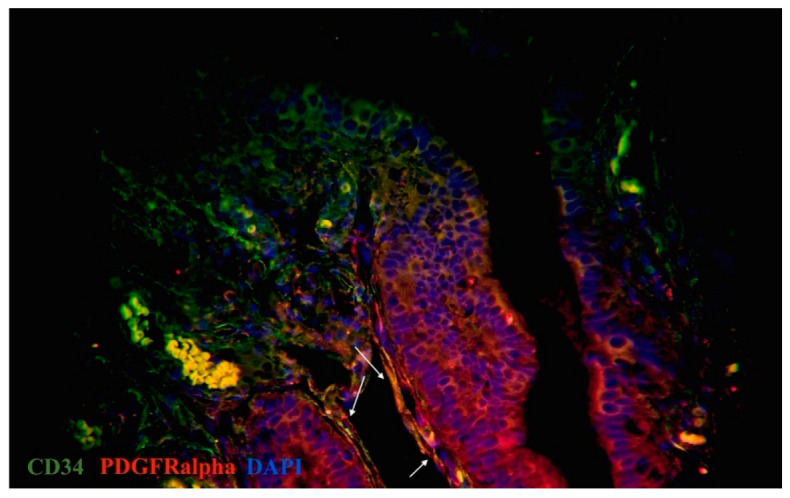
Double immunolabelling of human oviduct stained for PDGFRα (red, Alexa Fluor 594) and CD34 (green, Alexa Fluor 488) in study group. Nuclei stained by DAPI. Doubly immunopositive cell with an oval-shaped body and long cellular prolongation identified as telocytes (marked by arrows). Total magnification: ×400.

**Figure 7:**
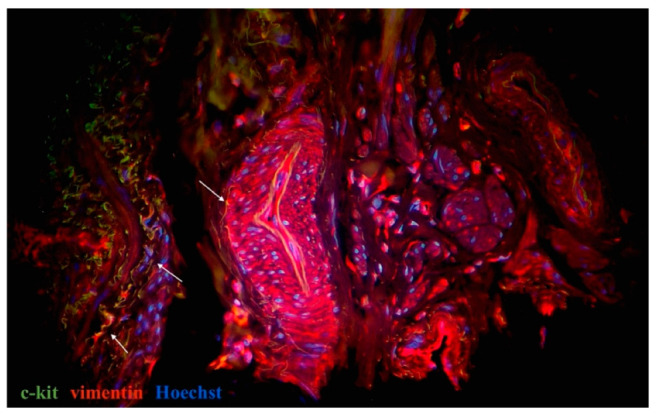
Tissue samples from human oviduct from control group stained for vimentin (red, Alexa Fluor 594) and c-kit (green, Alexa Fluor 488). Nuclei stained by Hoechst 33342. Doubly immunopositive cell with oval-shaped body and long cellular prolongation identified close to blood vessels and within tunica muscularis–oviductal telocytes (marked by arrows). Total magnification: ×400.

On the basis of the current literature data and our previous results, CD34, c-kit, vimentin, and PDGFRα were used for the primary identification of uterine and oviductal telocytes. Nuclei were stained by DAPI or Hoechst. Oviductal and uterine telocytes have the same morphology (small oval-shaped body, from two to three long cellular prolongations, sometimes crossed by each) and were often located close to blood vessels of different calibres within myometrial fibres. The unchanged myometrium had more telocytes than the myoma, while the adjacent myometrial tissue (especially close to the capsule of a myoma) had more than in the fibroid foci, and less than in the healthy myometrium. The myoma was characterised by sharply decreasing, or sometimes even disappearing, telocytes (Figure 4 and Figure 5). The human oviducts from the study group had more telocytes than the control group (Figure 6 and Figure 7). This difference in expression was common for all the observed tissue samples. Of note, the uterus and oviduct telocytes were positive for oestrogen and progesterone receptors. Localisation close to blood vessels and myoma capsules explained its role in local oxygen homeostasis.

### 2.5. Expression of Oestrogen and Progesterone Receptors

The expression of progesterone and oestrogen receptors in oviducts, the unchanged myometrium, the foci of the fibroid, and the adjacent myometrium were detected by immunohistochemistry. All the types of observed tissue samples were positive for both sex steroid hormone receptors. Generally, the prevalence of expression was common for the mucosa of the oviduct; it has been rarely observed in the tunica muscularis. In patients with uterine myoma, the distribution of oestrogen and progesterone receptor expression in the oviduct was characterised by the declining expression of oestrogen receptors and rising progesterone receptors (significantly higher than in the control). The same tendency was common for the foci of myomas. The expression of oestrogen receptors was lower in the foci of fibrosis (within myomas) than in the unchanged myometrium from the control group. Myometrial tissue, located close to a myoma capsule (adjacent myometrium), was characterised by the poor expression of sex steroid receptors (compared to the foci of myoma and unchanged myometrium). The oviductal tissue from the control group had fewer progesterone receptors and more oestrogen receptors. By comparing the expression in both groups, we note that the control group was characterised by a prevalence of oestrogen receptors. However, the patients with uterine myoma had two common tendencies concerning its expression: increasing progesterone receptor expression and declining oestrogen receptors (compared to control). The unchanged myometrium (“healthy”) has more oestrogen receptors than all the observed samples from the study group (myoma and adjacent myometrium). In our study, we reveal that, in the study group, the pattern of sex steroid receptors retained the expression in the fibroid foci. We emphasise that no pathological changes were revealed in the oviductal tissue from the study group. The tubal and uterine telocytes were positive for both nuclear steroid receptors and were revealed in all observed tissue samples.

### 2.6. Biochemical Parameters

Both groups showed no difference in the FSH and LH blood levels, while the oestradiol and progesterone levels depended on the presence of uterine myoma. Patients with uterine myoma had higher levels of progesterone in the blood. Oestradiol was higher in the control group (Table 2). These data correlate with an expression of sex steroid hormones in the tissue samples. The level of AMH was lower in patients with uterine myoma. No significant differences in the blood levels of soluble VEGFR1 (sflt) were revealed in either group of patients; however, oviductal and myometrial tissue have different expressions of sflt, based on the group.

## 3. Discussion

Uterine myoma is characterised by the exceeded production of the extracellular matrix (ECM) and the formation of avascular areas. Some common myoma growth factors are overexpressed in fibrosis foci, compared to the surrounding myometrium—VEGF A had increased activity in uterine myomas [30]. Vascular endothelial growth factor is an endothelial-specific mitogen that is produced by macrophages, platelets, tumour cells, renal mesangial cells, and keratinocytes [31]. The interaction between VEGF and sex steroid hormones in the female reproductive system is dual. Oestrogens increased the expression of VEGF mRNA more than progestins in a rodent uterus [32]. The opposite thesis is presented by Wang et al., who provide evidence that 17β-oestradiol can suppress VEGF expression and angiogenesis in patients with triple-negative breast cancer [33]. In the oviduct, the VEGF-A expression depends on the anatomical location [19]. It might relate to the embryo transport to the uterine cavity or to extrauterine pregnancy development. In addition, VEGF is considered to be a main mediator of angiogenesis in the primate ovulatory follicle in the ovary [34], and it may be a new marker of the porcine-oocyte-maturation competence during in vitro culture [35]. The balance of VEGF isoforms controls follicle progression and luteogenesis. Qiu et al. hypothesised that the control of the VEGF isoform expression may regulate fertility in mammals [36]. Moreover, this growth factor may have a neuroprotective action, as previously described in the central nervous system [31]. Our results demonstrate that the oviducts from the patients with uterine myoma had less VEGF expression than the control group. On the basis of the above statements, this group of patients (at least only with uterine myoma) might have a risk of the anovulation and transport dysfunction of the oviduct.

**Figure 8:**
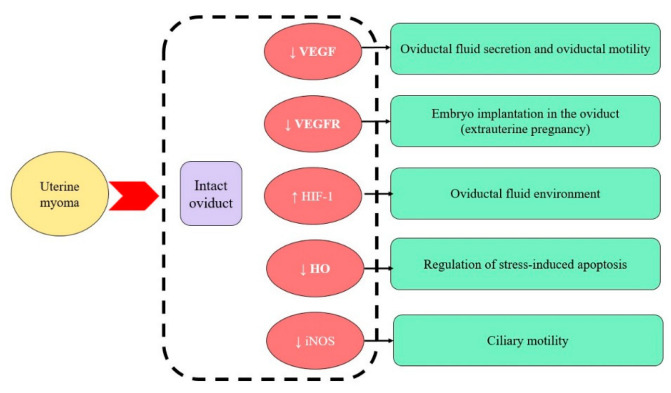
Diversity in oviductal oxygen homeostasis, caused by uterine myoma presence and its consequences in the context of fertility.

TCs could promote angiogenesis [37]. Aschacher et al. describe these cells in blood vessels and hypothesise that they might be involved in aortic tissue homeostasis by shedding microvesicles (sMVs) and exosomes containing angiogenic factors, such as VEGF—A [38]. Our results show that a declining number of myometrial and oviductal TCs in patients with uterine myoma correlates with a low expression of VEGF in the same samples of tissues. In the myometrium, this can be explained by the avascular areas in myomas and the specific angiogenesis on the borders with the adjacent myometrium, depending on the myoma size [26]. Nonetheless, the decline in TCs and the expression of VEGF was common for patients with uterine myoma, which might lead to local oxidative imbalance, oxidative damage, and mitochondrial dysfunction. This might result in oviductal contractility disorders and infertility development, which commonly coexist with uterine myoma in women. The expression of VEGF receptors is also lower in the oviducts of patients with uterine myoma (Figure 8). Normally, oviductal epithelial cells and oviductal stromal fibroblasts secrete VEGF and its receptor under hypoxic conditions. Itoh et al. suggest that this may contribute to the normal and pathological processes of oviductal fluid secretion by regulating the oviductal vascular permeability during the menstrual cycle [39], which is also important for fertility. Decreasing expressions of these proteins reflect the local oviductal environment.

Heme oxygenase-1 (HO-1) has a cytoprotective effect on cellular stresses and oxidative injury, and overexpression might also be connected to the progression of neurodegeneration and carcinogenesis [40]. HO-1 is usually connected to neurodegenerative diseases, cardiovascular diseases, cancer, metabolic diseases, iron-metabolism disorders, and various inflammatory diseases [41]. Liong et al. demonstrate that the HO-1 gene and protein expression were raised in terms of labouring myometrium, while, in nonlabouring myometrium, it was not [42]. In terms of labouring myometrium, it has been characterised by the overexpression of progesterone receptors and changes in the proinflammatory marker profiles [42,43]. Moreover, the progesterone gene expression was changed in the cultured myometrial tissue on the basis of the period of pregnancy [43]. The number of TCs in the myometrium also changes on the basis of the reproductive stage. Myometrial TCs decreased in the pregnant uterus and rose postpartum [44]. It has been reported that TCs constitute about 7% of the total cell numbers in nonpregnant myometrial cell cultures, and about 3% of the entire cell population in the myometrium of adult nonpregnant humans [44,45]. Previously, we showed a reduction in the number of TCs in the foci of myoma, compared to the adjacent myometrium and unaffected by uterus myoma [46]. The opposite tendency was common for oviductal TCs—in the oviductal tissue samples from patients with uterine myoma, we observed more telocytes than in the control group [23]. The difference in the number of cells in pregnant and nonpregnant uteri emphasises its involvement in the myogenic contractile mechanisms [47]. In addition, TCs are susceptible to hypoxia [47,48] and are involved in tissue repair. We focused our attention on HO-1′s role in oxidative stress and inflammation, observing a correlation with TCs. HO-1 is also associated with the regulation of the coagulation/fibrinolytic system. Patients with uterine myoma have oviducts with increasing amounts of telocytes and a lower expression of HO-1. We hypothesised that low HO-1 expression leads to a reduction in oxidation resistance, while rising telocytes might have a secondary regenerative compensatory function.

HIF-1 is expressed in all cell types and plays a role in the cellular response to hypoxia, immune-cell adaptation, the expression of immune genes, inflammatory processes, and immunosuppression in tumours [49,50]. Hypoxia may impact the oviductal and uterine fluid environments [51]. Luo et al. show that VEGF (HIF target gene) stimulates the fertilisation and maturation of bovine oocytes. In rats, hypoxia also impacts reproduction and fertility [51,52]. He et al. describe that the adaptation of reproduction in hypoxic conditions is associated with a greater expression of HIF-1α in the reproductive axis of female yaks [53]. The HIF-1 expression in oviductal tissue increased in patients with uterine myoma, reflecting accession to the pathology of the uterus disorders in the oviduct.

On the basis of our former results, we summarise that the normal (unchanged) myometrium has a higher prevalence of oestrogen receptor expression than progesterone expression, while, in the uterus affected by myoma, the balance between the steroid hormone receptors was changed. The myoma was characterised by a prevalence of progesterone receptors. This also correlates with a high progesterone level in patients with uterine myoma. The expression of oestrogen receptors was lower in the myoma than in the unchanged (“healthy”) myometrium. In the myometrium adjacent to areas of unchanged myometrium, which were close to a myoma capsule, the expression of both types of receptors was decreased (vs. that in normal and myoma-affected tissue samples) [24]. The oviductal tissue in patients without myoma has more oestrogen receptors, while uterine myoma has a prevalence of progesterone receptors (within the foci of fibrosis and the adjacent tissue) [23]. TCs have receptors for both steroid hormones. As previously mentioned, oestrogen can stimulate VEGF secretion, and TCs secrete it. The increasing progesterone receptor expression and oviductal telocyte rise lead to decreasing VEGF production in the oviduct. We also stress that the hormonal balance in our patients’ blood did not wholly reflect the presence of myoma (the exception is progesterone); the expression of the markers and hormones in the tissue samples depended on it, and the number of telocytes. This demonstrates the importance and necessity of focusing attention on tissue homeostasis, even at the beginning of disease development.

We are aware of the limitations of this research, which has a predominantly observational character, as we assessed histological samples posthysterectomy. Thus, the possibility of providing a deep analysis of patients’ medical records in the context of the infertility/fertility status was limited. Thus, the presented data partially support the conclusions regarding the direct link between the oviductal telocyte density and infertility. However, immunolabelling revealed that oviductal oxygen homeostasis is changed upon the presence of uterine myoma.

## 4. Materials and Methods

### 4.1. Subjects

The study group was comprised of 14 patients with uterine myoma (51.8 ± 6.3 years), while the control group was comprised of 15 patients without uterine myoma (49 ± 10.3 years) who underwent elective surgery for other reasons, and who had no pre- or intraoperative signs of uterine myomas. All women undergoing a laparoscopic hysterectomy were enrolled in the current study. Hysterectomy was performed according to the standard procedure. Tissue samples from the ampullar part of both oviducts (left and right) from all patients with and without uterine myoma were taken for further observation. Furthermore, tissue samples from the fibrotic foci and adjacent myometrium were obtained from the study group for further observation. Samples of unchanged myometrium were also obtained from the control group. In total, 58 oviducts were observed in the current study. Postsurgical histological examination of the uterus and fallopian tubes did not reveal any signs of malignant tumours.

### 4.2. Ethical Approval

The study was conducted in accordance with the moral, ethical, regulatory, and scientific principles governing clinical research. All the surgical samples were retrieved with the approval of the Jagiellonian University Bioethical Committee, using procedures that conformed to the guidelines of the Declaration of Helsinki (Protocol No. 1072.6120.48.2018).

### 4.3. Tissue Processing

Fresh hysterectomy specimens were collected and rinsed thoroughly with PBS (phosphate-buffered saline, 0.01 M, pH = 7.4), fixed in 4% phosphate-buffered paraformaldehyde, routinely processed, and embedded in paraffin. Serial sections were cut and mounted on poly-L-lysine-coated glass slides.

### 4.4. Routine Histology

The sections were deparaffinised, rehydrated, and stained with haematoxylin–eosin (H&E) to evaluate the gross tissue organisation.

### 4.5. Immunofluorescence

After deparaffinisation and rehydration, the slides were incubated for 30 min in PBS with the appropriate normal serum and 0.3% Triton X-100 (Sigma, St. Louis, MO, USA) at room temperature, followed by overnight incubation at 4 °C in a solution of PBS with the appropriate normal serum containing a primary antibody (or a mixture of primary antibodies) and 0.3% Triton X-100. After 5 washes (10 min each) in PBS, the specimens were incubated for 1 h at room temperature with a secondary antibody (or a mixture of secondary antibodies) and diluted in PBS with the appropriate normal serum and 0.3% Triton X-100. Finally, the slides were washed in two changes (10 min each) of PBS and cover-slipped with a fluorescence mounting medium (Dako, Glostrup, Denmark). Some slides have DAPI nuclear counterstain from UltraCruz^®^ Aqueous Mounting Medium with DAPI (catalogue number sc-24941) or Hoechst 33342 nuclear counterstain (Thermo Scientific, catalogue number 62249). Labelled specimens were analysed immediately. The following primary and secondary antisera were used (Table 3).

### 4.6. Microscopic Examination

Slides were examined using an MN800FL epifluorescence microscope (OptaTech, Warszawa, Poland) equipped with an Olympus DP74 digital CCD camera. Digital images were collected at 200×, 400×, or 600× magnification. The qualitative analysis of cells and nerve fibres was provided in 10 consecutive high-power fields of vision (400×) using the Multiscan 18.03 (CSS, Warsaw, Poland) computer-based image-analysis system. All samples were assessed by two independent specialists (each blinded to the other) who had no knowledge of clinical parameters or other prognostic factors to avoid bias.

Nerve cells and fibres were evaluated on the basis of their morphology. iNOS immunoreactivity was evaluated to assess the presence and distribution of different populations and subtypes of autonomic nerves. For primary identification of TCs, we used three widely used and proven immunohistochemical combinations of markers, as specified in the literature [23,29,44]. TCs were considered as cells that were double-positive for CD34/ PDGFRα and c-kit/vimentin, which is typical for this type of cell morphology and localisation. Moreover, CD34-positive interstitial cells lacked CD31 immunoreactivity, thus distinguishing them from CD34-positive/CD31-positive vascular endothelial cells and identifying them as TCs. We also combined double immunostaining with nuclei staining to denote the cellular nature of telocytes.

Expression of hypoxic markers and proangiogenic factors was evaluated by analysing HO, HIF-1, VEGF, and sFlt-1 (VEGFR-1) in all tubal and myometrial tissue samples (including the foci of myoma). Additionally, immunopositivity for oestrogen and progesterone receptors was compared in all the tissue samples. We repeated this immunostaining and compared it with our former results.

### 4.7. Biochemical Analysis

Blood samples from the medium cubital vein (5 mL) were collected in plastic tubes and incubated for at least 30 min at 4 °C to induce clot formation. After centrifugation at 1500× *g* for 20 min at 4 °C (Megafuge 1.0R, Heraeus Instruments, Germany), serum samples were separated and kept frozen in small volumes at −20 °C until further analysis. Samples were thawed immediately before the assays. All measurements were performed in duplicate. Luteinizing hormone (LH), follicle-stimulating hormone (FSH), oestradiol, progesterone, and anti-Mullerian hormone (AMH) levels were determined in the blood serum using photometric assays measured with Roche/Hitachi Cobas 6000/e601, Roche/Hitachi Cobas PRO/e801, and Cobas 8000 analysers.

## 5. Conclusions

Uterine and tubal dysfunction are crucial in female fertility. Even in cases untouched by pathological processes, human oviducts might have a local hormonal imbalance, a unique hypoxic profile, and differences in cellular compounds. Our results demonstrate that the population of oviductal telocytes grew in patients with uterine myoma, while they were significantly decreased in the fibroid. Moreover, the expression of sex steroid receptors in the oviduct reflects its change in the myoma foci. The oviduct in patients without myoma was more sensitive to VEGF and its receptor, but it had a lower expression of hypoxia-inducible factor-1, which, in turn, stimulates NOS production. These changes in morphologically healthy oviducts lead to disturbances in oxygen homeostasis, which might be a reason for infertility development. We are convinced that the mentioned oviductal modification, as well as the rising telocyte density, which accompanies the uterine myoma, should be assumed and deeply observed due to the influence of the fertility status.

## Figures and Tables

**Table 1:** Relative frequencies of observed angiogenic markers in the myometrial and oviductal tissue from the study and control groups. 0 = absence; (+) = very few; + = few; ++ = moderate density; +++ = multiple density.

	Uterus without Myoma		Uterus with Myoma		
	Myometrium	Oviduct	Myoma	Adjacent Myometrium	Oviduct
VEGF	++	++	(+)/+	+++	+
VEGFR (sFlt-1)	+	+++	++	++	++
HIF-1	++	+	(+)	+++	++
HO-1	+++	++	+	++	+

**Table 2:** Biochemical analysis of the blood samples collected from 29 patients (both the control and study groups). Data expressed as mean ± SD.

Parameter	Control Group	Study Group
AMH (pmol/L)	23.49 ± 14.84	6.48 ± 3.63
sflt (pg/mL)	87.31 ± 14.03	82.44 ± 6.47
FSH (mIU/mL)	57.63 ± 29.3	62.4 ± 39.63
LH (mIU/mL)	29.62 ± 15.15	33.83 ± 23.25
Oestradiol (pmol/L)	141.1 ± 253.86	96.68 ± 126.8
Progesterone (nmol/L)	0.32 ± 0.16	10.00 ± 14

**Table 3:** Type, sources, and dilution of antibodies.

Antibody	Catalog Number and Company	Dilution
**Primary Antibodies**		
Polyclonal goat anti-PDGFR alpha	AF-307-NA, R&D Systems, Minneapolis, Minnesota, USA	1:100
Polyclonal goat VEGFR1/FLT-1	AF 321, R&D Systems, Minneapolis, Minnesota, USA	10 µg/mL
Monoclonal mouse anti-human VEGF	Clone VG1, M7273, Dako, Glostrup, Denmark	1:100
Polyclonal rabbit anti-VEGFR-1	orb127531, Biorbyt, UK	1:100
Polyclonal rabbit anti-c-kit	A4502, Dako, Glostrup, Denmark	1:100
Monoclonal mouse anti-CD31	C70A, Dako, Glostrup, Denmark	1:100
Monoclonal mouse anti-CD34	M7165, Dako, Glostrup, Denmark	1:100
Monoclonal mouse anti-HIF-1	ab16066, Abcam, Cambridge, UK	1:100
Polyclonal rabbit anti-heme oxygenase 1	ab13243, Abcam, Cambridge, UK	5 µg/mL
Monoclonal mouse estrogen receptor	NCL-L-ER-6F11, Leica Biosystems, Newcastle upon Tyne, UK	1:50
Monoclonal mouse progesterone receptor	Clone PgR636, Dako, Glostrup, Denmark	1:100
Polyclonal mouse anti-NOS	sc-7271, Santa Cruz, Dallas, Texas, USA	1:100
Monoclonal rabbit anti-CD34	ab81289, Abcam, Cambridge, UK	1:200
Monoclonal mouse anti-vimentin	Clone V9, Dako, Glostrup, Denmark	1:50
**Secondary antibodies**		
Alexa Fluor 594 Donkey Anti-Goat	705-585-003, Jackson ImmunoResearch, Ely, UK	1:400
Alexa Fluor 594 Goat Anti-Mouse	115-585-146, Jackson ImmunoResearch, Ely, UK	1:400
Alexa Fluor 488 Goat Anti-Rabbit	111-545-144, Jackson ImmunoResearch, Ely, UK	1:400
Polyclonal Swine Anti-Rabbit FITC	F0205, Dako, Glostrup, Denmark	1:40
Alexa Fluor 488 Goat Anti-Mouse	115-545-146, Jackson ImmunoResearch, Ely, UK	1:400
Alexa Fluor 488 Donkey Anti-Goat	705-545-003, Jackson ImmunoResearch, Ely, UK	1:400
Alexa Fluor 488 Rabbit Anti-Mouse	315-545-045, Jackson ImmunoResearch, Ely, UK	1:400

## Data Availability

Data are available from the authors upon reasonable request.
